# Investigating the Relationship between HbA1c Level and Dental Caries in Type 2 Diabetic Patients: Relationship between HbA1c Level and Dental Caries in Diabetic Patients

**DOI:** 10.31661/gmj.v13iSP1.3738

**Published:** 2024-12-31

**Authors:** Ali Firouzi, Mehrdad Rajabbi

**Affiliations:** ^1^ Department of Periodontology, Dental Faculty, Shiraz University of Medical Sciences, Shiraz, Fars, Iran

**Keywords:** Type 2 Diabetes, HbA1c, DMFT Index, Dental Caries

## Abstract

**Background:**

Diabetes Mellitus is among the most frequent enduring metabolic disorders in the broad populace and leads to many complications, including tooth decay. In this study, the relationship between HbA1c level and dental caries has been investigated to be a step towards increasing awareness and improving the health status and quality of life of diabetics.

**Materials and Methods:**

This cross-sectional study was carried out on 20 type 2 diabetic who referred to dental clinic of shiraz university of medical sciences between 2022 and 2023 and were separated into two categories of managed diabetes (HbA1c under 7%) and uncontrolled (HbA1c above 7%). The desired data were obtained through a questionnaire containing demographic information, clinical examination (DMFT (Decayed, Missing, Filled Teeth) index), oral hygiene, and carbohydrate consumption.

**Results:**

The average DMFT index is greater in individuals with unmanaged diabetes compared to those with Controlled Diabetes, though not statistically significant (P=0.137). However, as BMI increases, so does the DMFT index (P=0.035). There is no notable correlation between HbA1c levels and dental caries (P=0.2). Oral hygiene practices did not significantly affect the DMFT index (P=0.943). Carbohydrate intake per day did not impact the DMFT (P=0.34). Higher education levels were associated with a decrease in DMFT, though not statistically significant (P=0.172).

**Conclusion:**

Considering the higher rate of dental caries in individuals with uncontrolled diabetes, it is necessary to inform these patients about more oral and dental hygiene and blood sugar control.

## Introduction

Diabetes Mellitus is one of the most frequent long-term metabolic disorders in the overall populace, marked by suboptimal blood sugar regulation. Among these, type 2 diabetes (T2D) is the predominant form of diabetes, comprising over 90% of all diabetes instances [1].

The cause of type 2 diabetes mellitus is both environmental and genetic factors, and this condition is caused by incomplete secretion of insulin and environmental resistance to insulin (decreased sensitivity of insulin receptors). The statistics of the World Health Organization show that the quantity of individuals enduring diabetes globally has escalated from 108 million persons in 1980 to 422 million persons in 2014, and most of these people live in developing countries [2][3].

Obesity, inactivity, family history, race, age, gestational hyperglycemia, and multicystic ovarian syndrome are predisposing elements for non-insulin-dependent diabetes mellitus [4][5]. One of the criteria for diagnosing and controlling diabetes and preventing diabetes complications is measuring the glycated hemoglobin (HbA1c) level. The level of HbA1c indicates the blood glycemic history of the past 120 days. It should be measured for every diabetic patient at 3-month intervals to determine the status of diabetes management and glycemic control. HbA1c level less than 7% is considered ideal in diabetic patients [6][7]. Diabetes leads to many complications and problems in the mouth. Certain research has indicated that oral and dental diseases in diabetic patients are 2-4 times more than the normal population. Multiple studies demonstrate that the frequency, advancement, intensity, and scope of persistent oral ailments in individuals with diabetes escalate considerably [8].

In addition to the other complications mentioned, the primary oral issues linked to diabetes encompass gum inflammation, periodontal disorders, dental caries, bacterial and fungal infections, and extended healing times following dental procedures [9]. Also, due to low saliva and increased saliva sugar concentrations because of serious insulin lack, individuals with diabetes are in great peril of tooth decay and decay advancement [10].

In general, the most common scale and one of the key scales to evaluate the state of dental caries in dental epidemiology is the DMFT index, which is the total number of decayed permanent teeth (D), missed due to caries (M) and filled due to caries (F) [11][12]. As previous studies have investigated the relationship between HbA1c levels and dental caries in type 2 diabetic patients with larger sample sizes, but often overlooked the regional specifics and demographic characteristics of the population, we aimed to explore this relationship in a specific context, namely in Shiraz, Iran, where this association has not been previously well examined. Furthermore, our study seeks to contribute to the existing literature by providing information about the oral health status and dental caries experience of type 2 diabetic patients in this region, which may have distinct environmental, genetic, and lifestyle factors that influence the relationship between HbA1c levels and dental caries.

## Materials and Methods

This project was approved by the Ethics Committee of Azad Shiraz Dental School and also after explaining the purpose and nature of the research to the study subjects, the consent form was obtained from all the participants.

### Study Type and Sample Size

This research was conducted as a cross-sectional study. The sample size was powered according to the prevalence of diabetes in the community (11.5%), the number of 20 affected patients was determined and they were examined according to the inclusion and exclusion criteria. The inclusion criteria for the study were as follows: patients between the ages of 30 and 65 years who had been diagnosed with type 2 diabetes and had the approval of a specialist. Additionally, patients must have had a disease duration of at least one year and at least one remaining natural tooth. The exclusion criteria included patients with type 1 diabetes, those with complications such as cardiovascular disease, renal impairment, or liver disease, patients using medications known to affect glucose metabolism or oral health (such as corticosteroids, immunosuppressants, or bisphosphonates), and those undergoing chemotherapy or radiation therapy.

### Study Procedure

Before entering the patients, all surfaces of the office were disinfected and the patient was examined as the first patient who entered the office. Then the tests related to the blood sugar of the patients were checked and the level of HbA1c related to three consecutive periods was checked and its average was calculated. These 20 patients were divided into two groups; According to ADA criteria, patients with HbA1c greater than 7% were in the uncontrolled diabetes group, and patients with HbA1c equal to and less than 7% were in the controlled diabetes group. Then, a questionnaire including two sections was designed. The first part included demographic variables (age, gender, education, duration of diabetes, number of sugary foods consumed per day, oral and dental hygiene practices, height, and weight) and the second part included BMI and DMFT index, which is the number of teeth decayed, missing and filled teeth were examined and noted for each patient and the DMFT number for each patient was calculated from the sum of the number of decayed, filled and missing teeth.

For the DMFT index, a comprehensive oral examination was conducted by a trained and calibrated dentist. The dentist used a dental mirror and explorer under adequate lighting conditions to ensure precise and consistent measurements. In this study, the remaining teeth, extra teeth and wisdom teeth were not included in the study and the DMFT index was calculated from among 28 permanent teeth. The standard for identifying the state of teeth concerning decay, restoration, and loss was based on the guidelines established by the World Health Organization. In this way, whenever damage was seen on the smooth surfaces of the teeth or in the pits of the tooth, in which the under enamel was empty or the bottom and around the damage was softened, it was considered as decay (D). The teeth that were missing only due to caries were counted as missing (M). A tooth that had permanent or temporary filling on one or more surfaces but had no decay, or when the restoration was defective but no old or new decay was observed in it, was considered as a filling tooth (F).

HbA1c levels were measured using a high-performance liquid chromatography (HPLC) method, by the NayoCard HbA1c Reader (Abbott, made in Germany). Blood samples were collected in EDTA tubes and analyzed within 24 hours of collection to ensure accuracy. Both the HPLC instrument and the dental examination tools were regularly calibrated to maintain high standards of reliability and validity.

To assess the oral hygiene practices of the participants, a structured questionnaire was administered. The questionnaire included a specific section on oral hygiene methods, with participants asked to indicate their primary method of oral hygiene from the following options: "None," "Toothbrush," and "Toothbrush and dental floss."

To evaluate the frequency of carbohydrate consumption, participants were asked to report how many times they consumed carbohydrates per day. The questionnaire provided the following options: "1 time," "2 times," "3 times," and "4 times or more." The data were collected through face-to-face interviews conducted by trained research assistants.

### Statistical Analysis

After collecting the data, classification and coding were done. The data were analyzed using non-parametric tests, Kruskal-Wallis (mean of several independent groups), Mann-Whitney (mean of two independent groups) and Spearman’s correlation coefficient in the SPSS version 24 and the level of P<0.05 was considered significantly.

### Ethical Statement

This project was approved by the Ethics Committee of Azad Shiraz Dental School.

## Results

**Table:** Table1. Descriptive Information of the Main Research Variables

**Variables**		**Frequency (percentage)**
**HbA1C**	HbA1C<7	9 (45%)
	HbA1C>=7	11 (50%)
	None	2 (10%)
**Oral hygiene**	Toothbrush	10 (50%)
	Toothbrush and dental floss	8 (40%)
	1 time	4 (20%)
**Consuming carbohydrate per day**	2 times	5 (25%)
	3 times	9 (45%)
	4 times or more	2 (10%)

**Table:** Table2. Mean DMFT Test between Two HbA1c Groups

**Variables**	**Number**	**Max-Min**	**Mean**± **SD**	**P-value**
**HbA1C <7**	9	18-5	10.22±4.206	0.137
**HbA1C>=7**	11	22-5	13.55±5.203	

**Table:** Table3. DMFT Mean test Based on Oral Hygiene Practices

**Variables**	**Number**	**Max-Min**	**Mean**± **SD**	**P-value**
**None**	2	13-11	12±1.414	
**Toothbrush**	10	20-5	11.9±5.425	0. 943
**Toothbrush and dental floss**	8	22-5	12.25±5.339	

**Figure-1:**
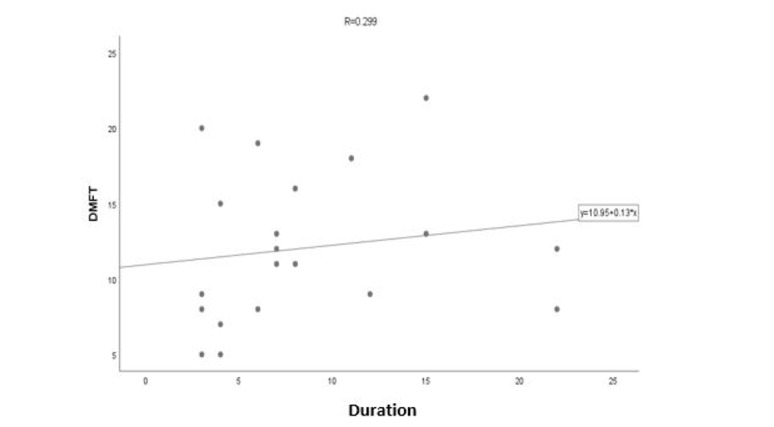
Figure 1. Distribution of DMFT in relation to duration of diabetes. The drawn line is very inclined to the horizontal line. This line shows that there is a direct and very weak relationship between two variables

**Figure-2:**
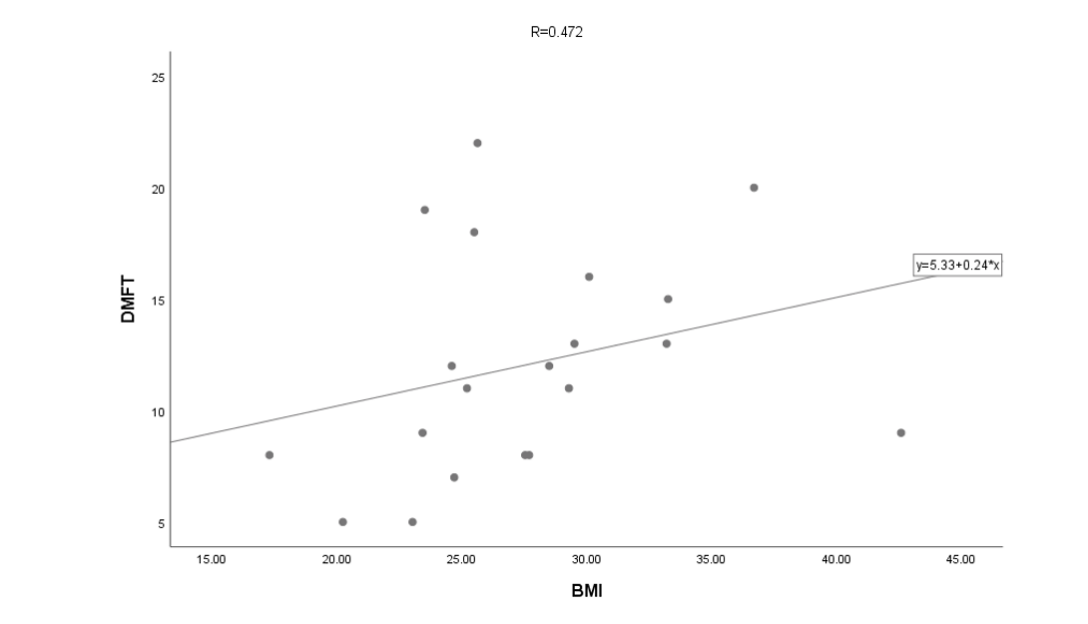
Figure 2. Dispersion of DMFT in relation to BMI index

In this research, the variables of gender, age, and education were included as demographic variables. The frequency of each variable is summarized in Table-1. In all the samples, the ratio of men and women was the same (50%) and most of the patients (60%) were in the education level of diploma to bachelor. The youngest patient in the sample was 40 years old and the oldest patient was 74 years old, and the average age of the studied patients was 55.65. According to Table-2, there is no significant difference between the average DMFT between the two groups (P=0.137). The minimum DMFT in both groups is 5 and the maximum is 22 in the HbA1c uncontrolled diabetes group and 18 in the HbA1c controlled diabetes group. Although this result does not show a significant difference, the average DMF in the HbA1c uncontrolled group (13.55) is higher than the controlled group (10.22).

In addition, the results of examining the average DMF in the HbA1c between men (11.7±5.417) and women (12.4±4.719) based on the Mann-Whitney test did not confirm a significant difference between the two groups (P=0.544).

Moreover, the average DMFT in the sub-diploma group is higher than the other three groups. The average in this group is 13.8 and with the increase in the level of education, the average DMFT gradually decreases. So that the average DMFT in the diploma to bachelor group is 12.33 and in the master education group and above it is reported as 8. There was no significant difference in the mean of DMFT in the study groups (P>0.05).

Kruskal-Wallis test was performed in order to check the significance of DMFT average based on the number of times of carbohydrate consumption per day. Based on the results, the frequency of carbohydrate consumption does not affect the average DMFT (P=0.34). In the group with the characteristics of 3 times carbohydrate consumption, the average DMFT was reported as 14.22 and more than the other two groups. After that, the highest average is assigned to the group with 1 consumption (11.25). In the third place is the group with 2 times of consumption with an average of 10.4 and at the last group of consumption 4 times and more with an average of 8.

In Table-3, the results of the significance analysis of the average amount of DMFT based on oral and dental hygiene practices are shown. Since the value of sig=0.943>0.05 was calculated, the result of the test is not significant and the mean of DMFT is not significantly different based on the way of observing oral and dental hygiene. The maximum amount of DMFT was reported as 22 among patients who use dental floss and toothbrushes. The average in this group is also higher than the other two groups (12.25). Among the patients who do not observe any hygiene, DMFT changes are insignificant (minimum is 11 and maximum is 13). The average in this group is reported to be 12.In the studied patients, a direct correlation between the duration of the disease and the amount of DMFT was observed at the rate of 0.299, which means that as the duration of the disease increases, the amount of DMFT also increases. But the results of the test do not confirm the significant relationship (P=0.2, Figure-1). In addition, Spearman’s correlation coefficient test does not show a significant relationship between the two variables of people’s age and the level of DMFT (sig=0.05>0.088). In the research sample, a direct relationship of 0.392 between two variables has been reported. Based on this, the sample values ​​show that DMFT increases with age.

Among the studied patients, a direct relationship with the magnitude of 0.472 was calculated between the BMI index of the subjects and the amount of DMFT. This value means that with the increase in the BMI index of people, the amount of DMFT also increases (sig=0.05<0.035, Figure-2).

## Discussion

The main aim of this study was to find the relationship between poor diabetes control and DMFT index in type 2 diabetic patients. According to the data analysis, the findings indicated that the average DMFT index in the group of individuals with uncontrolled diabetes (HbA1c≥7%) are higher than those with controlled diabetes (HbA1c <7%), but this variation is not statistically meaningful.

In contrast to our study, which found no statistically significant difference in DMFT index between patients with controlled and uncontrolled diabetes [13][14][15], previous research has consistently demonstrated a significant association between poor diabetes control and higher DMFT index values [14][15]. For instance, a study published in the Ups J Med Sci found that the number of decayed teeth and DMFT index values were significantly correlated with serum HbA1c levels [13]. Similarly, another study published in the Indian J Public Health reported a significant association between dental caries and HbA1c levels in adults with type 2 diabetes mellitus [14]. Furthermore, a nationwide Korean survey revealed that individuals with elevated blood sugar demonstrated a greater incidence of gum disease and increased tooth decay scores in contrast to the comparison cohort [15].

The result of the present study is in agreement with the results of studies by Aziz Khalid et al,.[13], Collin et al.[14], Rajaee et al,. [15], and Yonekura et al,.[16]. But the results of the studies of Majbauddin et al,.[17], Farahat et al,.[18], Jawed et al,.[19], Stojanovic et al,. [20] are contrary to the results of this study. It is possible that the number of people studied is different, the presence of very old people in some studies, the criteria for entering and excluding the study, which are effective in limiting the effect of confounding factors, are different in different studies. The reason for some of their results being inconsistent with the results of the present research.

Many factors such as the presence of caries-causing microorganisms, diet, immune and health factors of the patient are effective in causing dental caries in a certain period of time, although chronic infections and inflammatory diseases (such as dental caries) lead to an increase in blood glucose and HbA1c increases [21].

In the present study, with the increase in the BMI index, DMFT also increased significantly, which aligns with the findings of the research by Song et al. (23) and contrasts with the outcomes of the investigation by Majbauddin et al. [17] and Yonekura et al. [16]. The reason for the relationship between these two indicators can be the change in people’s lifestyle, the consumption of more snacks and drinks containing carbohydrates, processed and high-calorie foods, which are associated with a decrease in the flow of saliva and at the same time cause obesity and caries. But if people have a high level of health, obesity will not necessarily lead to an increase in DMFT [22].

In our study, there is no significant relation between disease duration and increasing DMFT score. Based on several studies, with an increase in blood sugar, saliva sugar increases, pH and saliva flow decrease, and the amount of DMFT also increases. but it is possible that a diabetic patient has been infected for a longer period of time, but his blood sugar is very controlled and his health is very good, so it cannot be said definitively that the duration of infection as an independent factor plays a strong role in causing caries because caries is a multifactorial disease, and hygiene, the number of caries-causing microorganisms in the oral environment, and other factors are involved in its development [23][24].

In addition, in the present study, no significant relationship was observed between the DMFT index and the way of oral and dental hygiene, education level, number of carbohydrate consumption per day and gender.

One of the strengths of this study is limiting and minimizing the effect of confounding factors by determining the criteria for entering and exiting the study more precisely than other studies and separately investigating the relationship of factors such as age, gender, oral hygiene, education, obesity, and the number of times of carbohydrate consumption per day is with DMFT, which adds to the value and importance of the research. According to most studies, we expect DMFT to be lower, by considering the average of several HbA1c courses for each patient, we increased the accuracy of the research, which was not done in previous studies.

One of the most notable constraints of this investigation is the minimal participant count of merely 20 individuals. This restricted number of subjects diminishes the analytical strength of the study, making it challenging to identify substantial variations and connections. The minimal participant count might also constrain the applicability of the results to a wider group. Subsequent research with more extensive participant counts is necessary to validate the initial patterns noted in this study.

In conclusion, this study showed that DMFT was greater in individuals with poorly managed blood sugar than in those with well-managed diabetes, although the relationship was not statistically significant. The inference derived from this research highlights the necessity to instructing individuals with poorly managed diabetes regarding enhanced oral and dental cleanliness and superior blood glucose regulation, as these elements might possibly decrease the occurrence of tooth decay.

## Conflict of Interest

The writers affirm no dispute of interest.
